# In vivo assessment of TiO_2_ based wear nanoparticles in periprosthetic tissues

**DOI:** 10.1007/s00216-024-05320-x

**Published:** 2024-05-10

**Authors:** Filip Gregar, Jiří Gallo, David Milde, Jitka Hegrová, Pavla Kučerová, Jakub Grepl, Tomáš Pluháček

**Affiliations:** 1 Department of Analytical Chemistry, Faculty of Science, Palacký University Olomouc, 17. listopadu 12, Olomouc, 771 46 Czech Republic; 2 Department of Orthopaedics, Faculty of Medicine and Dentistry, Palacký University Olomouc, University Hospital Olomouc, I. P. Pavlova 6, Olomouc, 77520 Czech Republic; 3 Transport Research Centre, Division of Sustainable Transport and Transport Structures Diagnostics, Líšeňská 33a, Brno, 619 00 Czech Republic

**Keywords:** Inductively coupled plasma mass spectrometry, Fractionation, Titanium nanoparticles, Tissue samples, Scanning electron microscopy, Raman spectroscopy

## Abstract

A multimodal approach combining inductively coupled plasma mass spectrometry (ICP-MS), single-particle ICP-MS (spICP-MS), scanning electron microscopy-energy-dispersive X-ray spectroscopy (SEM-EDS) and Raman spectroscopy enabled a deeper insight into the balance between total titanium (Ti), the soluble titanium fraction and titanium dioxide based particle fraction levels in periprosthetic tissues collected from patients undergoing revision surgery. Hydrofluoric acid usage in the sample digestion allowed for complete digestion of TiO_2_ particles, thus enabling accurate estimation of total Ti levels. The TiO_2_ fraction represents 38–94% of the titanium load in the six samples where particles were detected, and the fraction is present mainly in samples from patients with aseptically loosened total hip arthroplasty. Further attention was given to this fraction determining the elemental composition, particle count, particle size and modification of TiO_2_. The spICP-MS analysis confirmed the presence of the TiO_2_-derived (nano)particles (NPs) with a 39- to 187-nm median size and particle count up to 2.3 × 10^11^ particles per gram of tissue. On top of that, the SEM-EDS confirmed the presence of the TiO_2_ nanoparticles with 230-nm median size and an anatase crystal phase was determined by Raman spectroscopy. This study presents a novel multimodal approach for TiO_2_ particle determination and characterization in tissue samples and is the first *in vivo* study of this character.

**Supplementary Information:**

The online version contains supplementary material available at 10.1007/s00216-024-05320-x.

## Introduction

Metals and their alloys are widely used to manufacture orthopaedic or dental implants [1]. Notably, titanium and titanium based alloys are considered superior for hip joint implants thanks to their excellent biocompatibility and mechanical and biotribological features, including a passive thin TiO_2_ layer protecting the surface from corrosion. The total hip arthroplasty (THA) represents the only and thus routine surgery for patients with advanced end-stage hip joint diseases or trauma. The estimated average number of THAs implanted in the European Union just before the COVID-19 pandemic was around 168 per 100,000 population, ranging from 50 (Turkey) to 315 (Germany) per 100,000 population. Importantly, it could almost double the figures in some countries with data from private hospitals [2].

Historically, many THAs used during the last 30 years have consisted of shells from Ti-6Al-4V with polyethylene/ceramic liner and stems from Ti-6Al-4V. Several mechanisms of degradation of metallic implants have been described to date [3], including titanium alloys [4]. Once THA is placed in a body, it is subjected to an inevitable degradation, which is the collective effect of corrosion, fatigue, and wear [5]. Together, these processes lead to the release of ionic metals and nanoparticles (NPs) from the implant and its oxide film into the periprosthetic tissue which may result in adverse health issues either systemic or local (aseptic loosening, soft tissue pathology, allergic reaction, pain etc.) [6–8]. Some studies have suggested pro-carcinogenic potential [9], neurotoxicity [10], reprotoxicity [11] and possible health risks to offspring growing inside mothers exposed to TiO_2_ [12].

The *in vitro* studies revealed that the TiO_2_ nanoparticles are produced as an interaction with natural biological media [13, 14]. In addition, a medium containing a mucosal bacterium *Streptococcus mutans* was able to affect the parameters of released particles. Accordingly, diameters of released particles ranged from 10 to 160 nm and the authors demonstrated the possibility of smaller particles entering osteoblast cells [15]. Another recent study reports TiO_2_ nanoparticles might influence osteoblast derived exosome cargos affecting by this way osteogenic differentiation of human mesenchymal stem cells [16]. Taken together, the information on the total metal amount in periprosthetic tissues may be insufficient to assess the potential health implications. Further examination of the abundance and size of potential particles should be carried out to investigate the issue completely. Moreover, when using the approach of solution analysis of tissue samples, the presence of an oxidic form of titanium in samples requires a modification of the digestion mixture to be completely dissolved. This is usually done by the addition of hydrofluoric acid [17, 18] into the digestion mixture or digestion with sulfuric acid [19]. The widely used digestion mixture composed of nitric acid (HNO_3_) or its combination with hydrogen peroxide (H_2_O_2_) can provide underestimated values, especially for periprosthetic tissues with higher levels of nanoparticle fraction. The comprehensive analysis of TiO_2_ derived nanoparticles released from failing hip/knee replacements providing unique information on size distribution, particle abundance and crystal structure has not been established yet. X-ray fluorescence spectroscopy (XRF) and X-ray absorption near-edge structure (XANES) have been successfully applied to identify metallic and TiO_2_ forms in inflamed human tissues around dental implants [20]. Recently, the *in vitro* study has been focused on the determination of size distribution and particle abundance of released TiO_2_ derived particles from titanium based dental implants using a single-particle inductively coupled plasma mass spectrometry (spICPMS) in combination with transmission electron microscopy [15]. The spICP-MS approach has already been utilized in the hunt for various kinds of nanoparticles differing in size, chemical composition and concentration in many fields of interest. The spICP-MS is preferred due to the simultaneous and accurate determination of dissolved and particular forms of transitional metal or its oxide along with counting and sizing of NPs fraction [21, 22]. On top of that, accurate determination of titanium and TiO_2_ derived nanoparticles at trace levels remained an analytical challenge even in the case of using triple quadrupole ICP-MS instruments.

In this work, we have studied the occurrence of titanium and its fractions released into the periprosthetic tissue of 21 patients undergoing revision of their failed THA/total knee arthroplasty (TKA) by a multimodal approach. This approach includes the digestion of the sample with modified acid mixtures allowing the distinction between soluble ion titanium fraction and insoluble particle TiO_2_ fraction. Moreover, the spICP-MS method, scanning electron microscopy-energy-dispersive X-ray spectroscopy SEM-EDS and Raman spectroscopy were implemented to characterize the size, elemental composition, abundance of TiO_2_ NPs and its crystal structure. To the best of our knowledge, we are providing the first evidence of the TiO_2_ NPs presence *in vivo w*ith a detailed characterization of released nanodebris.

## Materials and methods

### Chemicals

Nitric acid (69%, Analpure®), hydrogen peroxide (30%, analytical grade+) and hydrofluoric acid (50%, analytical grade) obtained from Analytika Ltd. (Czech Republic) were used for sample digestion. ASTASOL® Titanium (1000 ± 2 mg/L) certified reference material purchased from Analytika Ltd. (Czech Republic) was used for calibration; TraceCERT® Titanium standard for AAS (1000 ± 4 mg/L) obtained from Sigma-Aldrich (USA) was used to prepare QC samples. Validation of the measurement procedure was performed using Titanium(IV) oxide nanopowder (21 nm) obtained from Merck KGaA (Germany). Alfa Aesar Gold nanoparticles, 40 nm, supplied in 0.1 mg/mL sodium citrate with stabilizer obtained from (Thermo Fisher, USA) were used for calibration of spICP-MS mode. All solutions were prepared using ultrapure water (18.2 MΩ cm) from Milli-Q purification system (Millipore Corporation, France). Chicken breast meat obtained from a local supermarket was used to validate and develop the proposed methodology.

### Samples

Samples from 21 patients located at our archive of retrieved periprosthetic tissues were used in the study, 19 patients had a hip replacement containing titanium (P1–P19), and two patients with THA/TKA from alloy not containing titanium were used as a control group (C1–C2). The summarized clinical data for all patients are presented in Table 1 (for details, please see Electronic Supplementary Material Table S1). Periprosthetic tissue samples were collected during revision surgeries at the Department of Orthopedics, University Hospital in Olomouc, Czech Republic. A variable volume of periprosthetic membranes was taken per each revision from the inner surface of the THA pseudocapsule in dependence on the degree of tissue hypertrophy seen by the surgeon. A size of a sample varied between 2 × 2 × 0.2 and 2 × 10 × 1.0 cm. Samples were placed directly into clean plastic containers in the operating room, and stored at −80 °C. Before the multimodal analyses, the periprosthetic tissues were lyophilized to dryness using a GREGOR L 10–55 PRO freeze dryer (Gregor Instruments, Czech Republic) and homogenized by milling on a ceramic coffee grinder.

**Table 1:** Summarized clinical data for patients

Characteristic	Patients (*n* = 21)
Sex	16 female 5 male
Implant type	19 titanium based (patient group) 2 cobalt-chromium based (control group)
Time in situ	2.4–27.6 years
BMI	20.7–37.3
Number of revision surgery	1^st^ (*n* = 15) 2^nd^ (*n* = 5) 4^th^ (*n* = 1)

### Multimodal approach for the determination of soluble and wear particle forms of titanium in periprosthetic tissues

**Fig. 1:**
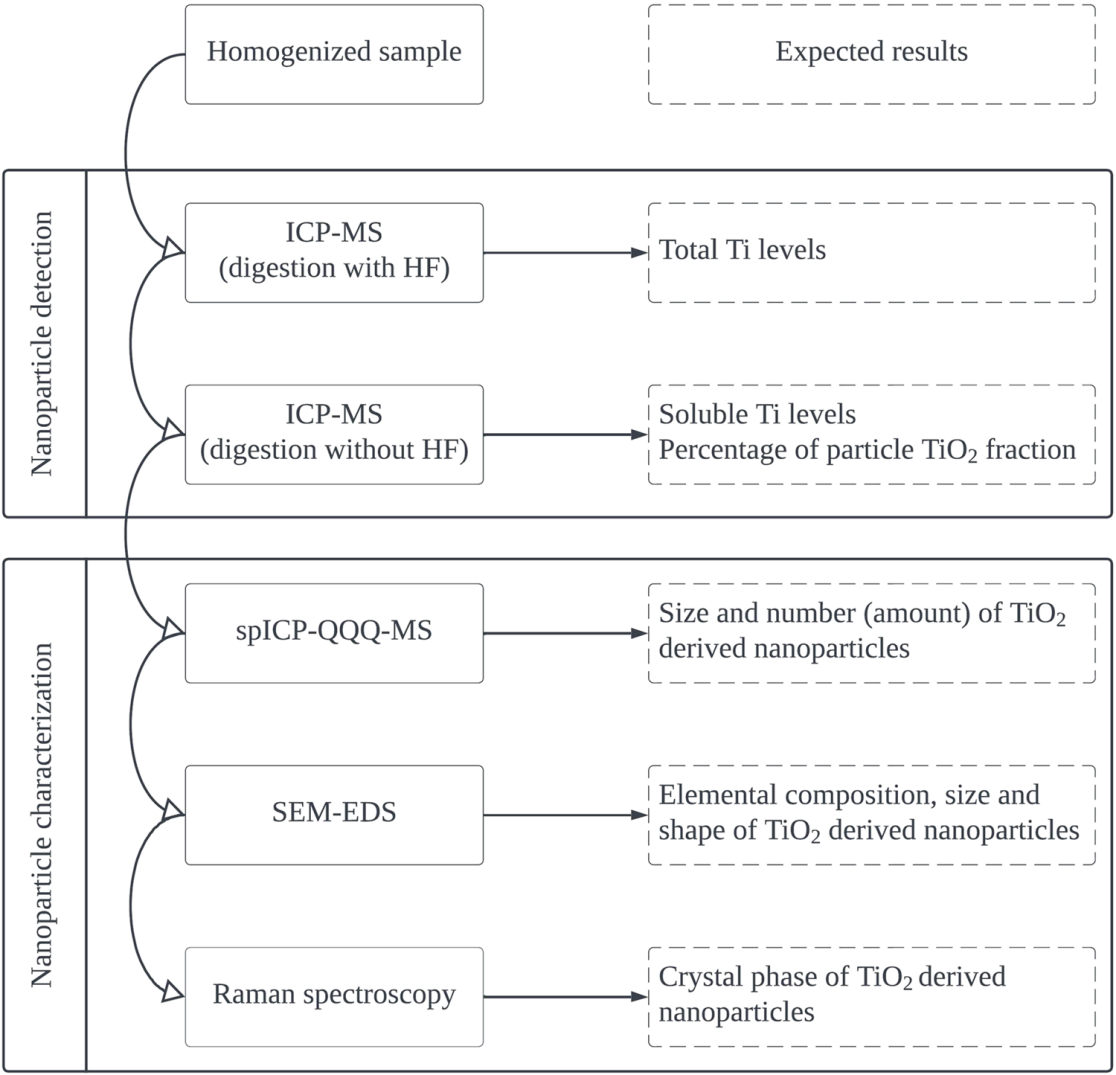
Scheme of the multimodal approach used for the elucidation of the titanium nature in the periprosthetic tissues (spICP-QQQ-MS: single-particle mode triple quadrupole ICP-MS, SEM-EDS : scanning electron microscopy-energy-dispersive X-ray spectroscopy)

The multimodal approach benefits from the combination of (i) ICP-MS method employing microwave digestion using hydrofluoric acid decomposing TiO_2_, (ii) ICP-MS method employing microwave digestion without hydrofluoric acid, (iii) spICP-MS method, (iv) SEM-EDS and (v) Raman spectroscopy. The scheme showing the method sequence and the outcomes is presented in Fig. 1.

### ICP-MS fraction analysis

#### Determination of total titanium

The microwave-assisted digestion using a mixture containing hydrofluoric acid was used to completely dissolve tissue samples. Approximately 50 mg of homogenized sample was accurately weighed into a Teflon vessel, and the mixture of 4 mL of ultrapure water, 1.5 mL of HNO_3_, 0.5 mL of H_2_O_2_ and 0.05 mL of HF was added. The sample was decomposed according to 8 steps power-controlled digestion program (2 min/250 W, 2 min/0 W, 5 min/400 W, 2 min/0 W, 2 min 500 W, 2 min/0 W, 6 min/600 W with 10-min cooling cycle at the end of digestion) in a Milestone MLS 1200 Mega microwave digestion system (Milestone, Italy). After cooling to a laboratory temperature, the digest was diluted using ultrapure water to 12–15 g, mixed, and transferred to the cleaned polypropylene test tube. Blanks were prepared by digestion without a sample matrix. The total Ti content was accessed by ICP-MS using an ORS-ICP-MS 7700x (Agilent Technologies, Japan). The ICP-MS instrument is fitted with an autosampler, micromist nebulizer, cooled Scott-type spraying chamber, and octopole reaction system working in He mode (minimize polyatomic interferences). The optimized ICP-MS parameters were as follows: a RF power of 1550 W, plasma gas flow rate of 15.0 L/min, an auxiliary gas flow rate of 0.9 L/min, a nebulizer gas flow rate of 1.05 L/min, a collision gas He flow rate of 4.3 mL/min and a dwell time of 200 ms for ^46^Ti, ^47^Ti and ^48^Ti and 100 ms for ^72^Ge (internal standard) isotopes. The quantitative data (^47^Ti isotope) were estimated by 10-point external calibration (0.5–10,000 µg/L), and the reliability of the ICP-MS results was controlled by a regular measurement of an independently prepared quality control sample (concentration levels of 100 and 500 µg/L prepared by serial dilution of TraceCERT® Titanium standard for AAS). All samples were measured in six technical replicates.

#### Determination of soluble titanium fraction

The samples for the determination of soluble titanium fraction were prepared by the same procedure from the previous chapter using a digestion mixture without HF. The insolubility of TiO_2_ NPs in digestion mixtures without HF allowed the extraction of soluble titanium species (ionic, bound to the proteins, etc.) originating from the tissue matrix and leaving the TiO_2_ based NPs intact. In order to separate the insoluble TiO_2_ fraction, two 2-mL aliquots of the final solution were transferred into a 2-mL Eppendorf tube and centrifuged at 16,000 g for 25 min at 4 °C. Afterwards, 0.8 mL of supernatant was taken from each Eppendorf tube, mixed and diluted to 4 g. Blanks were prepared by the same procedure without sample matrix. The soluble Ti levels were obtained by analysis on the same ICP-MS instrument described in the previous section.

#### ICP-MS method validation

The measurement procedure for determining total Ti levels, soluble Ti and Ti in the TiO_2_ NPs fraction was validated in terms of linearity, limit of detection (LOD) and limit of quantification (LOQ), trueness and precision in accordance with the FDA guideline on the validation of bioanalytical methods [23]. Linearity was assessed using Pearson’s correlation coefficient from the 10-point external calibration. Method LOD and LOQ were calculated as the standard deviation from the 6 blanks multiplied by 3 for LOD and by 10 for LOQ. Finally, the instrumental LOD and LOQ were recalculated using a sample weight and the sample dilution factor to get the LOD and LOQ in µg/g. Due to the lack of the appropriate certified reference material, the trueness (expressed as a recovery in %) and precision (expressed as an RSD in %) were validated on chicken meat spiked with TiO_2_ NPs solution at concentration levels of TiO_2_ of 74.9 µg/g (level 1) and 1298.7 µg/g (level 2) each prepared and measured in six replicates. The efficiency of centrifugation was verified by centrifuging a suspension of TiO_2_ NPs in water at concentration levels of TiO_2_ of 74.9 µg/g and 1298.7 µg/g, each prepared and measured in six replicates. The efficiency was expressed as a percentage of removed particles from the solution

#### Measurement uncertainty estimation

Measurement uncertainty of the results from ICP-MS determination of total Ti in periprosthetic tissue was estimated using the so-called top-down approach, where uncertainty was evaluated from validation data. Two major uncertainty components — precision (*s*_r_) and bias (*b*) — were combined using the law of propagation of uncertainties (Eq. 1) to obtain combined standard uncertainty (*u*_c_). The bias component was estimated from a spiking study described in the “ICP-MS method validation” section.

1 $${u}_{{\text{c}}}= \sqrt{ {s}_{{\text{r}}}^{2}{+ b}^{2}}$$

Finally, an expanded uncertainty (*U*) was calculated using the following equation *U* = *k*×*u*_c_, where *k* is the coverage factor and (*k* = 2) to present a confidence level of 95%. The results from both methodologies are expressed in the form of a mean ± expanded measurement uncertainty. Calculated uncertainty values cover contributions from the ICP-MS measurement and sample digestion. Contribution of sample heterogeneity could not be included into calculation due to the insufficient amount of periprosthetic tissues to perform detailed study of the heterogeneity.

#### Determination and characterization of TiO_2_ based nanoparticles

The samples suspected (6 patients) of the presence of TiO_2_ based nanoparticles were selected from the ICP-MS data. The presence (percentage) of TiO_2_ was calculated using the following formula (Eq. 2). 

2 $$\mathrm{insoluble}\;{\mathrm{TiO}}_2\;\mathrm{fraction}\;(\%)\;=\;\frac{\left(\mathrm{total}\;\mathrm{Ti}\;\mathrm{content}\;-\;\mathrm{soluble}\;\mathrm{Ti}\;\mathrm{content}\right)}{\mathrm{total}\;\mathrm{Ti}\;\mathrm{content}}\times100,$$

  where total Ti content (µg/g) is total Ti levels after the digestion with HF and soluble Ti content (µg/g) is total Ti levels after the digestion without HF. The same procedure described in the “Determination of soluble titanium fraction” section was used for the sample preparation but without the centrifugation step. The properly diluted and sonicated sample solutions containing nanoparticle fractions were subjected to the spICP-MS analysis using an 8800 ICP-QQQ-MS instrument (Agilent Technologies, Japan) fitted with parts for single-particle mode analysis to estimate the size and number of TiO_2_ based nanoparticles. A diluted standard of 40 nm golden nanoparticles (NPs) was used for transport efficiency determination and a stock solution of ionic Ti standard (1 µg/L) was used for external calibration. The optimized ICP-MS parameters were as follows: RF power of 1550 W, and a dwell time of 3 ms for ^48^Ti isotope with the mass shift to ^48^Ti^16^O^+^ using the reaction cell in oxygen mode with a flow rate of 10%. Instrument performance was tuned daily by analysing a solution of 21 nm TiO_2_ NPs in Milli-Q water. Particle concentration was approximated for TiO_2_ nanoparticles with a size of 21 nm and density of 4.23 g/cm^3^.

The scanning electron microscope (SEM) TESCAN VEGA with integrated energy-dispersive spectrometer II and Tescan Essence software (TESCAN, Czech Republic) were used for obtaining SEM images, comparative measurements of TiO_2_ nanoparticle size and elemental composition. The accelerating voltage and the beam current were set to 25 kV and 30 pA, respectively. To prevent agglomerate formation, the sample material from patient 9 was diluted with acetone (1:1) and sonicated for 20 min and 1 µL was deposited on a carbon conductive disc and was left to dry.

The crystal phase of TiO_2_ nanoparticles was elucidated by Raman spectroscopy using Raman microscope DXR 2 (Thermo, USA). A selected sample from spICP-MS analysis was preconcentrated by drying under a nitrogen stream. 10 µL of the preconcentrate sample was transferred onto a microscope glass and allowed to dry at room temperature. The Raman spectra were collected by a green laser working at 532 nm within a 1200–100 cm^−1^ range. The spectra were accumulated for 30 s with a laser power of 10 mW and a spectrograph aperture of 50 µm pinhole size. The anatase TiO_2_ nanoparticle powder with a size of 21 nm (Merck KGaA (Germany)) was used as a reference standard.

## Results and discussion

### ICP-MS methodology validation

The validation of both ICP-MS methods described in the “ICP-MS fraction analysis” section (digestion with and without the HF addition) covered the evaluation of LOD, LOQ, trueness, precision under the repeatability conditions, and centrifugation efficiency, to comply with a validation strategy given in the FDA guideline on the validation of bioanalytical methods. The validation parameters together with acceptance criteria and the validation results are presented in Table 2. All validation parameters meet the acceptance criteria and confirmed that the ICP-MS methodology is suitable for its purpose. Slight differences in LODs and LOQs between the two methods are probably caused by the addition of hydrofluoric acid that might not have been of the same purity as other chemicals used in the study. However, this difference is negligible concerning the total content of Ti in real samples reaching up tens of mg/g. The centrifugation efficiency of 72 and 77% might at first glance seem low; nonetheless, we were able to obtain the result very precisely (RSD under repeatability conditions 2% and 3%). Secondly, the particles in real samples could be an order of magnitude larger than the particles of 21 nm used in the validation; hence, it is likely that the centrifugation will be more efficient in the real samples. The relative combined standard uncertainty associated with measurement results was estimated and *u*_c_′ = 13.3%. Then, the relative expanded measurement uncertainty for 95% confidence level was *U*′= 26.6%.

**Table 2:** Method performance parameters considered in method validation

Total Ti ICP-MS methodology		
Validation parameter	Acceptance criterion	Results
Linearity, Pearson’s correlation coefficient R	*R* ≥ 0.99	*R* = 1.000
Method LOD (without HF)	-	0.2 µg/g
Method LOQ (without HF)	-	0.5 µg/g
Method LOD (with HF)	-	0.8 µg/g
Method LOQ (with HF)	-	2.7 µg/g
Trueness	Recovery 80–120%	96% (L_1_) 106% (L_2_)
Precision	RSD ≤ 20%	13% (L_1_) 11% (L_2_)
Centrifugation efficiency (recovery)	-	77% (L_1_) 72% (L_2_)
Centrifugation efficiency (repeatability)	RSD ≤ 20%	3% (L_1_) 2% (L_2_)

*L*_1_ = 74.9 µg/g and *L*_2_ = 1298.7 µg/g levels

### Detection and determination of TiO_2_ fraction in periprosthetic tissues

The sequential ICP-MS approach benefiting from different strengths of the digestion mixtures provided unique information on titanium forms in the control and patient’s periprosthetic tissue samples. The suggested approach revealed the levels of total Ti and soluble Ti fraction as well as the presence of particle Ti fraction with a percentage abundance (Table 3). The percentage of the particle Ti fraction was calculated as a difference between the total Ti and soluble Ti fraction levels, and the differences lower than the expanded measurement uncertainty (34%, *k* = 2.58, 99% confidence level) were marked as no particle fraction detected (n.d.).

**Table 3:** Determined values of total Ti content and Ti fraction content (Ti contents expressed as an arithmetic mean ± expanded measurement uncertainty, *k* =2). *n.d.*, not detected

Sample	Total Ti (µg/g)	Soluble Ti fraction (µg/g)	Particle Ti fraction (%)
P1	14.8 ± 3.9	10.2 ± 2.7	n.d.
P2	197.9 ± 52.6	47.4 ± 12.6	76
P3	455.0 ± 121.0	340.0 ± 90.4	n.d.
P4	94.2 ± 25.1	58.4 ± 15.5	38
P5	22.5 ± 6.0	11.3 ± 3.0	50
P6	147.3 ± 39.2	118.1 ± 31.4	n.d.
P7	26.1 ± 6.9	33.0 ± 8.8	n.d.
P8	39.0 ± 10.4	46.7 ± 12.4	n.d.
P9	71,665.0 ± 19,062.9	12,590.0 ± 3348.9	82
P10	46.0 ± 12.2	45.5 ± 12.1	n.d.
P11	46.9 ± 12.5	57.9 ± 15.4	n.d.
P12	23.4 ± 6.2	21.8 ± 5.8	n.d.
P13	63.4 ± 16.9	48.8 ± 13.0	n.d.
P14	65.2 ± 17.3	53.9 ± 14.3	n.d.
P15	22.7 ± 6.0	28.2 ± 7.5	n.d.
P16	81.0 ± 21.6	61.2 ± 16.3	n.d.
P17	363.7 ± 96.7	21.8 ± 5.8	94
P18	14.6 ± 3.9	11.5 ± 3.1	n.d.
P19	573.3 ± 152.5	67.6 ± 18.0	88
C1	2.9 ± 0.8	2.7 ± 0.7	n.d.
C2	5.2 ± 1.4	4.5 ± 1.2	n.d.

In comparison with the control group, the expected elevated titanium levels for the patient group were observed. The results confirmed the widely accepted theory that the process of tribocorrosion produces a considerable amount of wear particles that can accumulate in the surrounding tissue. A load of released metallic byproducts into the periprosthetic tissue is highlighted by total Ti levels obtained by the ICP-MS method utilizing a hydrofluoric acid to decompose all titanium based forms, especially TiO_2_ completely. In comparison with generally accepted digestion procedures without HF, we observed approximately 2–17 times increase in the total Ti levels reflecting the contribution of TiO_2_ based nanoparticles. These results confirm the necessity of the addition of an agent in the digestion mixture that can cleave the bonds forming the crystal structure of TiO_2_ and liberate the bonded titanium ions into the solution. Other commonly used digestion procedure with H_2_SO_4_ was not considered in this research due to high boiling point of this acid (338 °C) that would damage Teflon vessels under power-controlled operation of the microwave digestion unit. Moreover, the digestion with sulfuric acid is not widely accepted due to the insolubility of the sulphates and purity issues [24].

The results for the total Ti content cannot be adequately compared with the literature as no similar study utilizing digestion of TiO_2_ fraction with HF or H_2_SO_4_ has been, to our best knowledge, published so far. On the other hand, the concentration ranges for total Ti are similar to previously reported data by Kuba et al. [25], where the authors also address the heterogeneity of titanium levels across different samples, thus making it even more challenging to compare with results from other studies. This variability has been discussed previously [1], and it is also observed in our results. The origin of the observed variability might be associated with a distinct degradative mechanism that is either single strong or a combination of multiple factors [5, 26]. A heterogeneous group of factors potentially inducing implant surface pathology might also contribute to the deliberation of Ti byproducts from the surfaces of THA. A role might play factors like body mass index (BMI), the period of implant service, particular implant placement in relation to a particular pattern of implant loading, fatigue behaviour, corrosive resistance or even specific geometry of the implant like its shape, off-sets or neck characteristics making the occurrence of subclinical implant impingement easier [26–28].

A significant amount of titanium based particle fraction was detected in periprosthetic tissues collected from patients P2, P4, P5, P9, P17 and P19. The contribution of particle form to total Ti loading in the proximity to implants ranged between 38 and 94%. These samples were subjected to the sp-ICP-QQQ-MS analyses providing unique information about the nanoparticle size, the distribution and the number of nanoparticles. Interestingly, the total titanium levels did not correspond with the presence of particle fraction and the relatively high Ti loading could be caused by the soluble Ti forms/wear particles. Four of six of these patients were revised for aseptic loosening and the remaining one for recurrent dislocation of THA (see Table S1). It may lead to a conclusion that the observed fraction of Ti byproducts is associated mainly with a pure abrasive mechanism than with the other ones (e.g. adhesion, tribo-chemical reactions or surface fatigue) [3, 4, 26]. However, to date, there is limited knowledge on the true mechanism of generation of TiO_2_ nanoparticles or the rules explaining their fractions in total Ti loading. The ICP-MS based methodology can be used as a routine tool to explore total Ti levels or the presence of Ti particle fraction in the periprosthetic tissue samples, thus becoming a promising approach to further understand the relationship between Ti levels and implant failing.

### Characterization of wear titanium based nanoparticles by spICP-MS

The single-particle approach on ICP-QQQ-MS instrument benefited from superior analytical performance on the determination of titanium on the most abundant isotope ^48^Ti resulting in the detectability of TiO_2_ nanoparticles with a diameter starting from 8 nm (instrumental limitation calculated from the lowest transient signal that could be detected as a particle). The tissue digests were measured by sp-ICP-QQQ-MS after adequate dilution (total dilution factor ranged from 1000 to 100,000) to both minimize the probability of simultaneous detection of two nanoparticles and decrease the background soluble titanium deteriorating the detection of small nanoparticles. Determined medians of particle size and particle contents are shown in Table 4. The median diameter of the released NPs ranged between 39 and 187 nm whereas the number of particles reached up to 2.3×10^11^ particles/g of tissue. It is worth mentioning that the determined particle size of TiO_2_ nanoparticle by sp-ICP QQQ MS can be affected by a particle agglomeration not overcome by a sonication step [15]. Thus, the particle agglomerates can slightly impact the results (for details on size histograms, please see Electronic Supplementary Material Fig S1).

**Table 4:** Particle size medians and particle contents obtained by spICP-MS analysis

Sample	Median diameter (nm)	Particle content (#/g)
P2	61	7.5 × 10^7^
P4	55	3.1 × 10^8^
P5	39	2.1 × 10^8^
P9	187	2.3 × 10^11^
P17	93	1.5 × 10^8^
P19	98	7.2 × 10^7^

The particle content ranges between 7.2 × 10^7^ and 3.1 × 10^8^ particles per gram. The largest particles and the highest number of particles were established in sample P9 (the case of strong abrasive wear), whereas sample P5 was the sample with the smallest particles. The possible conclusion could be that the bigger the total Ti load in the tissue, the bigger particles are generated, which is supported by results from the rest of the samples. The spICP-MS results are in good agreement with the ICP-MS revealing a huge portion of Ti in the TiO_2_ form. However, comparing the particle content might be difficult since the particle size distributions are vastly different between samples, and for proper comparison, it would be necessary to look at individual sample size histograms. This in-depth size comparison is beyond this study’s scope, which aims to bring new analytical approaches for studying titanium particles released from joint implants.

Regarding periprosthetic titanium load, there is the question of the origin of titanium metallic products in the adjacent tissues in relation to a mode of THA failure. This can be examined using experimental *in vitro* and *in vivo* settlements [5, 29, 30], via characterization of metallic byproducts in the periprosthetic fluid, tissues and peripheral blood [31], and by examination of retrieved implants [32].

In addition to clinical observation, the analysis of particle shape and size might help to determine the degradative mechanism behind (mechanical, tribo-chemical etc.) suggesting that bigger particles could be deliberated from an implant surface because of a rough abrasive (including third body) or impact mechanism while smaller ones by rather a finer mechanism (fretting, adhesion wear and tribocorrosion). In fact, several methods could be applied to identify sources of small titanium alloy particle products in the THA environment other than those related to aseptic loosening involving complex pathology of head-stem modular junction and tribo-chemical reactions [5, 33, 34]. However, our data preclude contribution to the explanation of inter-case variability in total/particulate titanium load observed in our cases with the detailed analysis of the other surfaces of retrieved implants because that study was not done at the time of surgery and the implants are not available now. The clinical importance of Ti particle load on patient health should be intensively studied as it may affect the local and/or systemic biological environment [28, 35].

## Case study

**Fig. 2:**
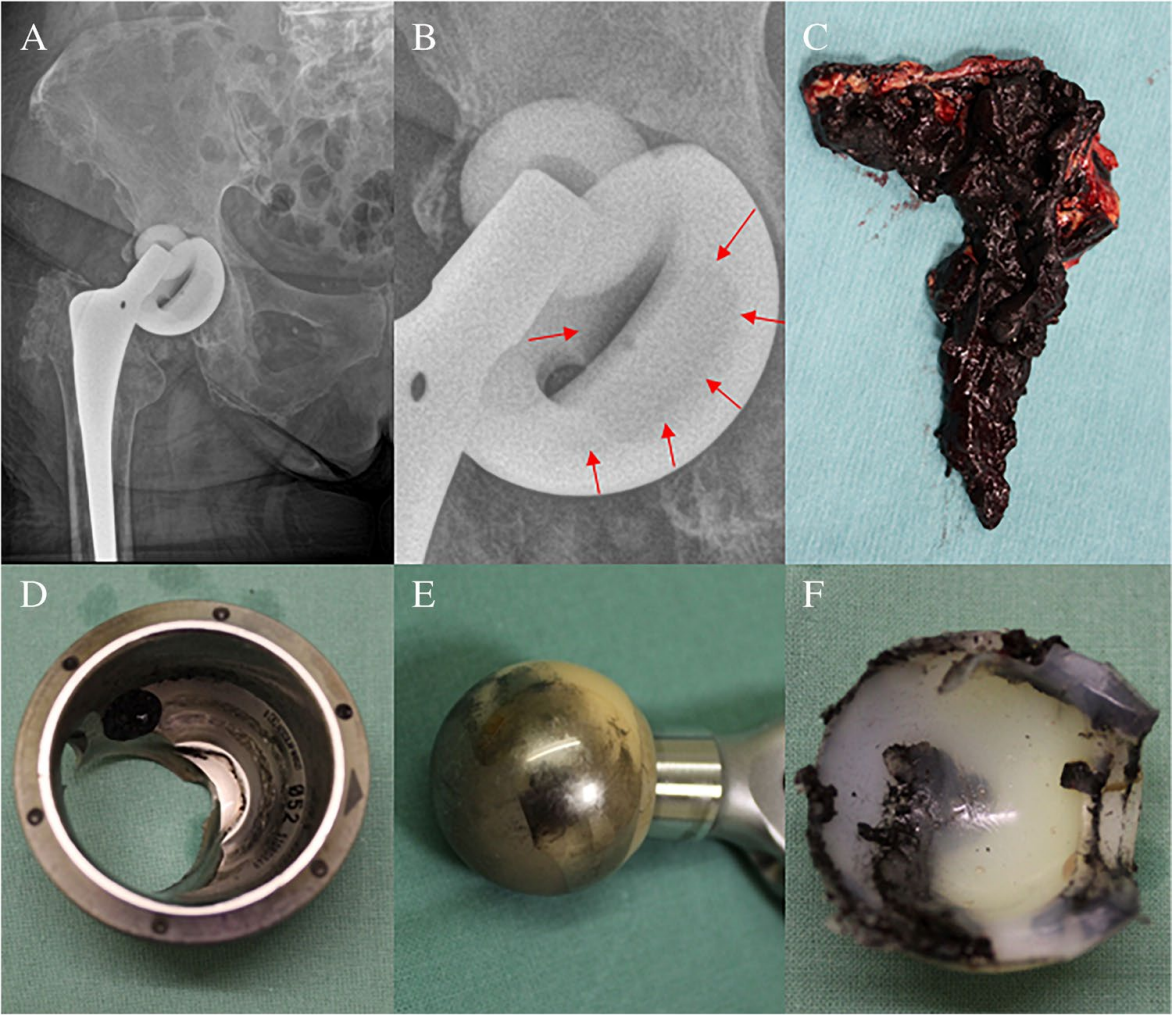
X-ray of the right hip of an 81-year-old female with a loosened cup (**A**) which is at some part wear-through (**B**); sample of periprosthetic tissues (**C**), the intraoperative picture of the removed metallic cup (**D**), ceramic head (**E**), and damaged polyethylene liner front side (**F**)

Lastly, the research focuses on a case study of tissue collected from patient P9 to get a deeper insight into the nature of titanium based wear particles released into the periprosthetic tissue. The clinical data are the following: female, at the time of index surgery 62 years old, BMI 33.1, primary osteoarthritis as the indication to THA, Plasmacup–Bicontact R implant (Aesculap, Germany): both the cup and stem were made from Ti alloy, 28-mm ceramic head articulated against polyethylene line. The time of the implant service was 19.1 years. It was the first revision, and the main reason for the reoperation was aseptic loosening, and wear-through of polyethene liner with massive metallosis (Fig. 2). We do not exactly know why this THA failed by this mode as hundreds of such combinations were implanted at the site of authors and even much more especially in Germany with relatively good 10-year results [36].

**Fig. 3:**
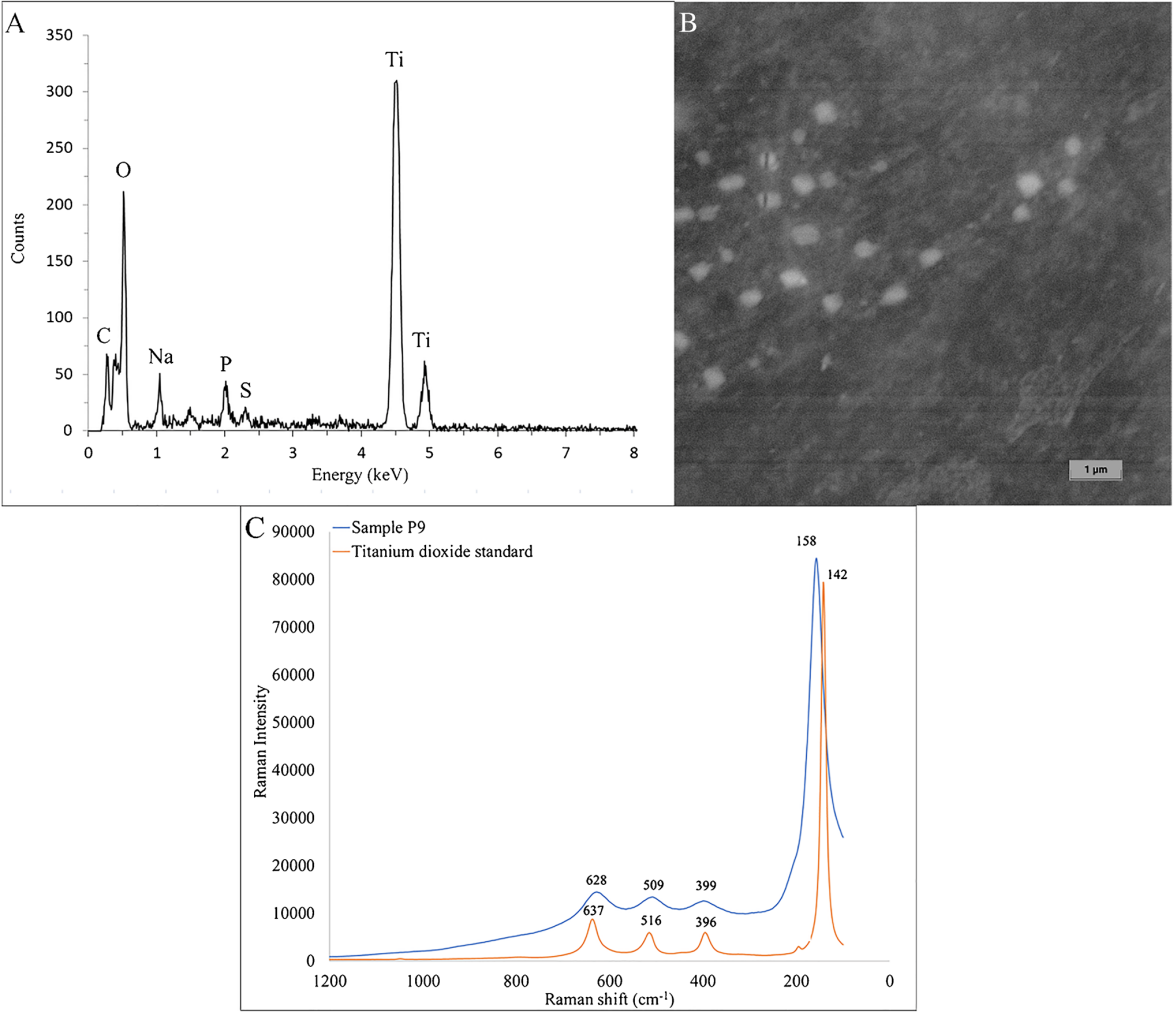
Characterization of released TiO_2_ nanoparticles isolated from the tissue of patient P9, EDS spectrum confirming the presence of TiO_2_ (**A**), SEM picture recorded using BackScatter detector (**B**) and Raman spectra for sample P9 and water suspension of standard 21 nm TiO_2_ nanoparticles (**C**)

The homogenized periprosthetic tissue was subjected to multimodal analysis to obtain the total levels of Ti, soluble Ti fraction and particle Ti fraction contents, particle elemental composition, size distribution and number of particles per gram of tissue as well as the crystal structure phase of TiO_2_. The titanium was presented mainly in the form of TiO_2_ derived nanoparticles with a size of 187 nm reaching approximately 82% of the total titanium content of 71,665.0 ± 19,062.9 µg/g. The nanoparticle size and the particle content of approximately 2.3 × 10^11^ particles per gram of lyophilized tissue were confirmed by spICP-QQQ-MS analysis of diluted sample digest (dilution factor of 100,000). The extensive spreading of the TiO_2_ derived nanoparticles into the periprosthetic tissue resulted in an extensive metallosis, which was visible by the naked eye. SEM-EDS analysis confirmed the presence of titanium dioxide in the sample. EDS spectrum shown in Fig. 3A indicates the presence of oxygen and titanium as evidence of TiO_2_ as well as carbon, phosphorus, sodium and sulphur which are expected as the sample is a digest of a biological matrix. The median size of the particles of 230 nm was established from the measurement of 177 individual particles on 5 consecutive samples. TiO_2_ nanoparticles in circular, oval, square, rectangular or triangular shapes of various sizes were observed (Fig. 3B). On top of that, the structural phase of the TiO_2_ (anatase, rutile and brookite) was studied using the Raman spectroscopy within a range of 1200–100 cm^−1^. From the Raman spectra in Fig. 3C, it is evident that the spectra have four distinct peaks, the first around 630 cm^−1^, the second around 510 cm^−1^, the third around 400 cm^−1^ and the last one being slightly shifted with a Raman shift of 158 cm^−1^ for the sample and 142 cm^−1^ for the TiO_2_ reference [37]. The presence of the characteristic shifts proved that the isolated particle Ti fraction is composed of TiO_2_ nanoparticles with an anatase structural phase.

The obtained data confirmed our hypothesis that hydrofluoric acid plays a crucial role in determining the reliable amount of released Ti from joint implants into the periprosthetic tissue. Moreover, its role in the complete digestion of TiO_2_ based nanoparticles could be used for purposes of fraction analysis, thus allowing the user of this methodology to distinguish between soluble and particle titanium fractions in the sample. The particle fraction content is several times higher when compared with the soluble fraction content. The SEM-EDS analysis is in agreement with the spICP-MS analysis, as it firstly confirmed the presence of TiO_2_ in particulate form and secondly affirmed the particle size measured by the spICP-MS. The slight difference in the measured median particle size (187 nm from spICP-MS and 230 nm from SEM) is probably the result of either the limited resolution of the SEM instrument or the spherical shape assumption of the spICP-MS approach.

The overall metallic load in the presented patient is the result of rough damage of the cementless titanium alloy cup caused by the ceramic head after a wear-through of the polyethylene liner. It takes a relatively long time to wear through the metallic shell because the Plasma cup has a thick metallic wall, thereby explaining the total amount of TiO_2_ particles observed in the periprosthetic tissues. In fact, such cases have been published previously [38–40]. However, to date, no study examined the size distribution of the particles deliberated from the surfaces of the titanium implants in THA, neither determined conditions affecting the proportion of nanoparticles on the total load of titanium byproducts. Unfortunately, TiO_2_ NPs are very insoluble persisting unchanged in the periprosthetic tissues for a long time [41]. Importantly, metallic submicron particles can not only stimulate macrophages and fibroblasts to pro-inflammatory response [42–45] but also have the capacity for transporting into distant biological compartments [46, 47].

### Study limitations

The new measurement methodology for establishing total Ti content, soluble Ti fraction and particle Ti fraction content was developed and successfully used to analyse Ti content in tissue samples of patients with THA. Nevertheless, it is essential to note some of the limitations of our approach. However, the methodology for such analysis has been proposed and validated. The titanium alloy implants contain other metals like aluminium and vanadium, and the whole artificial joint can also contain components made of cobalt-chromium alloys (metallic heads, stems), making it possible for the particles to contain these metals. Since the single-particle mode allows to measure only one element at a time, these “impurities” in analysed particles would go undetected or the metals might be dissolved in the digestion process leading to the breakdown of larger particles, thus distorting the results. Another limitation comes from the high ionic Ti content in our samples. The simultaneous analysis of ionic and particle element form is considered one of the most significant advantages of spICP-MS, although the high ionic Ti content results in the inability to detect small particles (the serial dilution steps were adopted to get a complex image of the released NPs size distribution). We cannot comment on the biological effect of the titanium byproducts as well as we cannot associate the measured amounts of titanium alloy byproducts with the reason of surgery because it was not the aim of the study. In addition, the detailed analysis of retrieved implant surfaces cannot be done because the implants are not available now.

## Conclusion

The proposed multimodal approach allowed the first *in vivo* detection and characterization of the released TiO_2_ derived nanoparticles in the periprosthetic tissue samples collected from patients with failed THA. On top of that, ICP-MS methodologies for the estimation of the levels of total Ti, soluble Ti fraction, and particle Ti fraction in samples of periprosthetic tissue have been developed, validated and used on real samples. This fraction analysis benefited from the microwave-assisted sample digestion with two different digestion mixtures, one containing a small amount of HF enabling the decomposition of the persistent TiO_2_ fraction. The addition of HF has enormously increased the total Ti levels, thus confirming the necessity of using the correct digestion mixture when assessing total Ti levels in the periprosthetic tissue of patients with failed THAs containing titanium based implants. The isolated titanium wear particles were characterized by the spICP-MS, Raman spectroscopy and SEM-EDS to get information of particle composition (TiO_2_), crystal phase (anatase), median size (39 and 187 nm) and count (particle count ranged from 7.2 × 10^7^ to 2.3 × 10^11)^. The release of the metallic byproducts seems to be associated with a reason of the revision surgery with stable implants producing lower amounts of Ti and vice versa. Moreover, the detailed case study performed on the samples collected from the patient undergoing first revision surgery of aseptically loosened THA revealed a huge amount of titanium and even 82% of released titanium was in the form of TiO_2_ derived nanoparticles with a median size of 187 nm (spICP-MS) and 230 nm (SEM-EDS). Our multimodal approach represents an important step in a deeper understanding of the potential health risks connected with the released TiO_2_ based particles from joint implants into the patient’s tissue. Further research in this area should examine periprosthetic Ti load on a larger group of patients with titanium THAs using the described analytical methods. This could improve our understanding of implant/patient-specific factors related to total Ti load of periprosthetic tissues, as well as the contribution of Ti particles to the adverse periprosthetic tissue response associated with periprosthetic inflammation, osteolysis and aseptic loosening. Additionally, Ti particles can act as abrasives, accelerating the wear rate of the THA. Furthermore, regulatory agencies in the EU and elsewhere are currently focusing on the potential health adverse effects of metallic byproducts, mainly cobalt based implants.

### Supplementary Information

Below is the link to the electronic supplementary material.

Supplementary file1 (DOCX 218 KB)

## Declarations

### Source of biological material

All of the biological samples were collected during revision surgeries performed at the Department of Orthopedics, University Hospital in Olomouc, Czech Republic, upon written informed consent of the included patients.
