# Supplementary material for: In vivo assessment of TiO2 based wear nanoparticles in periprosthetic tissues
Source: Anal Bioanal Chem. 2024 May 10;416(16):3785–96. doi: 10.1007/s00216-024-05320-x (PMC11180632; doi:10.1007/s00216-024-05320-x)
Supplement: Supplementary file 1 — Supplementary file1 (DOCX 218 KB) [file 216_2024_5320_MOESM1_ESM.docx]

**Supplementary file**

***In vivo* assessment of TiO_2_ based wear nanoparticles in periprosthetic tissues**

Filip Gregar^a^, Jiří Gallo^b^, David Milde^a^, Jitka Hegrová^c^, Pavla Kučerová^a^, Jakub Grepl^a^, Tomáš Pluháček^a*^

^a^Department of Analytical Chemistry, Faculty of Science, Palacký University in Olomouc, 17. listopadu 12, 771 46 Olomouc, Czech Republic

^b^Department of Orthopaedics, Faculty of Medicine and Dentistry, Palacký University Olomouc, University Hospital Olomouc, I. P. Pavlova 6, 77520 Olomouc, Czech Republic

^c^Transport Research Centre, Division of Sustainable Transport and Transport Structures Diagnostics, Líšeňská 33a, 619 00 Brno, Czech Republic

^*^Corresponding author

Correspondence should be addressed to:

Dr. Tomáš Pluháček

Department of Analytical Chemistry

Faculty of Science

Palacký University Olomouc

17. listopadu 12

771 46 Olomouc

Czech Republic

Phone: +420585634542

E-mail: tomas.pluhacek@upol.cz

1. **Patient’s clinical data**

The summarized clinical data for P1 – P19 and C1 – C2 are presented in Table S1. * metallic ball; # ceramic head; AL=aseptic loosening at least one component; BMI=body mass index; D=dislocation; M=metallosis; P=pain; W=polyethylene wear; PPOL=periprosthetic osteolysis

Table S1 Complete patient information

| Sample | Group | Implant | Material | Time in situ (years) | Height (cm) | Weight (kg) | BMI | Sex | Order of revision | Reason for revision | Primary diagnosis |
| --- | --- | --- | --- | --- | --- | --- | --- | --- | --- | --- | --- |
| P1 | Patient | Bicon-ABG* | Ti, Al, V, Co, Cr | 6.6 | 154 | 49 | 20.7 | Female | 2 | W, PPOL | Primary osteoarthritis |
| P2 | Patient | Allofit–SL stem* | Ti, Al, V, Co, Cr | 5.9 | 173 | 92 | 30.7 | Male | 1 | AL | Primary osteoarthritis |
| P3 | Patient | ABG-ABG* | Ti, Al, V, Co, Cr | 23.6 | 158 | 52 | 20.8 | Female | 1 | W, PPOL | Primary osteoarthritis |
| P4 | Patient | Plasmacup-SL+ stem# | Ti, Al, V, Nb | 19.5 | 183 | 125 | 37.3 | Male | 2 | AL | Post-Traumatic Arthritis |
| P5 | Patient | ABG-ABG* | Ti, Al, V, Co, Cr | 25.6 | 162 | 69 | 26.3 | Female | 1 | W, PPOL | Post-Traumatic Arthritis |
| P6 | Patient | Bicon-SL+ stem# | Ti, Al, Nb | 11.3 | 165 | 64 | 23.5 | Female | 1 | W, PPOL | Primary osteoarthritis |
| P7 | Patient | Balgrist-ABG* | Ti, Al, V, Co, Cr | 18.2 | 164 | 70 | 26.0 | Female | 2 | W, PPOL | Primary osteoarthritis |
| P8 | Patient | Trilogy-ACR* | Ti, Al, V, Co, Cr | 27.6 | 169 | 58 | 20.3 | Female | 1 | W, PPOL | Rheumatoid arthritis |
| P9 | Patient | Plasmacup-Bicontact R# | Ti, Al, V | 19.1 | 163 | 88 | 33.1 | Female | 1 | W, AL, M | Primary osteoarthritis |
| P10 | Patient | Allofit-Fitmore# | Ti, Al, V, Nb | 9.0 | 169 | 65 | 22.8 | Female | 1 | P | Post-dysplastic arthritis |
| P11 | Patient | Alloclassic-SL stem* | Ti, Al, Nb, Co, Cr | 6.6 | 167 | 87 | 31.2 | Male | 1 | W, P | Primary osteoarthritis |
| P12 | Patient | Balgrist-SL stem# | Ti, Al, Nb | 23.5 | 152 | 62 | 28.1 | Female | 1 | W, PPOL | Post-dysplastic arthritis |
| P13 | Patient | Bicon-ABG* | Ti, Al, V, Co, Cr | 10.8 | 177 | 94 | 30.0 | Male | 2 | W, PPOL | Primary osteoarthritis |
| P14 | Patient | Allofit-SL stem* | Ti, Al, V, Nb, Co, Cr | 2.4 | 167 | 74 | 26.5 | Female | 1 | P | Primary osteoarthritis |
| P15 | Patient | Bicon-Wagner# | Ti, Al, Nb | 13.3 | 168 | 88 | 31.2 | Female | 4 | P, W | Ischemic necrosis |
| P16 | Patient | Balgrist-SL stem# | Ti, Al, Nb | 21.4 | 170 | 90 | 31.1 | Female | 1 | W, PPOL | Primary osteoarthritis |
| P17 | Patient | Delta-PLS (Lima)# | Ti, Al, V | 9.5 | 162 | 89 | 33.9 | Female | 1 | AL | Primary osteoarthritis |
| P18 | Patient | Screw cup-Profemur# | Ti, Al, V | 8.3 | 179 | 81 | 25.3 | Male | 1 | W, PPOL | Primary osteoarthritis |
| P19 | Patient | Balgrist-SL stem# | Ti, Al, Nb | 20.5 | 152 | 65 | 28.1 | Female | 1 | W, PPOL, D | Primary osteoarthritis |
| C1 | Control | Exeter cup-Exeter stem* | Fe, Cr, Ni, Mn | 24.2 | 153 | 75 | 32.0 | Female | 1 | AL | Post-dysplastic arthritis |
| C2 | Control | Kinemax+ (knee) | CoCr alloy | 16.8 | 151 | 57 | 25.0 | Female | 2 | AL | Primary osteoarthritis |

1. **Single particle mode ICP-MS**

The TiO_2_ nanoparticles were characterized by the single particle ICP-MS method using an 8800 ICP‑QQQ-MS instrument equipped with a nanoparticle module to access the nanoparticle size and number/amount. The optimized ICP-MS parameters were as follows: RF power of 1550 W, and a dwell time of 3 ms for ^48^Ti isotope with the mass shift to ^48^Ti^16^O^+^ using the reaction cell in oxygen mode with a flow rate of 10%. Instrument performance was tuned daily by analysing a solution of 21 nm TiO_2_ NPs in MiliQ water. Particle concentration was approximated for TiO_2_ nanoparticles with a size of 21 nm and density of 4.23 g/cm^3^. The results of the spICP-MS analysis provided a size median of 36 nm (Figure S1, panel A), slightly differing from the size of 21 nm claimed by the manufacturer, which may be caused by the nanoparticle aggregation or the detection of multiple particles at the same time, these double signals are viewed by the instrument as particles with double mass. This way the size distribution shifts slightly to the bigger particles and the median is slightly larger than the reference size. The particle concentration of 4.2 × 10^7^ particles per litre resulting from our measurements is in accordance with our calculated theoretical value of 4.9 × 10^7^ particles per litre. For standard nanoparticles, it is reasonable to think about them as ball-shaped objects; thus, our calculations should reliably reflect reality. From the measurement of standard 21 nm TiO_2_ nanoparticles, it was assessed that the instrument was tuned properly. The TiO_2_ nanoparticle size histograms are presented in Figure S1, panel B-G. The median size for the TiO_2_ derived nanoparticles ranged between 39 – 187 nm.


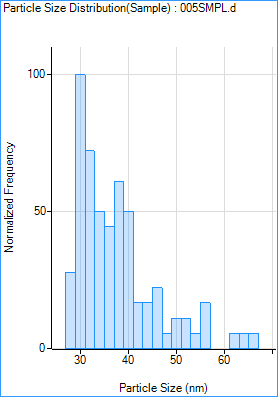

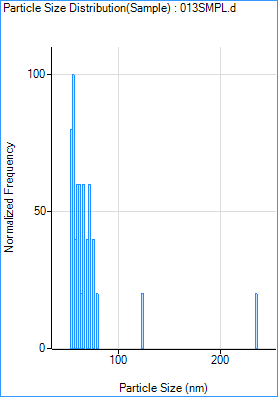

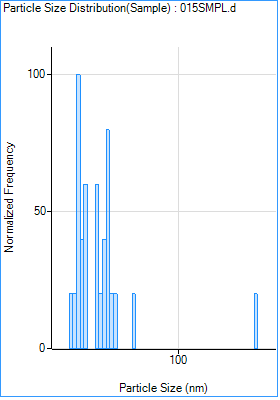

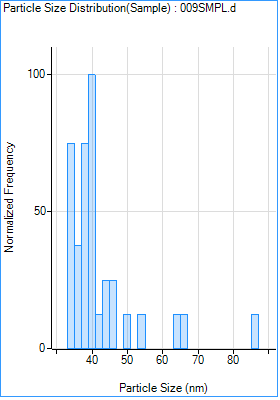

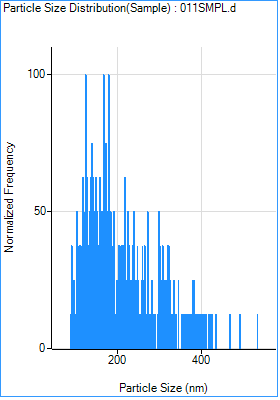

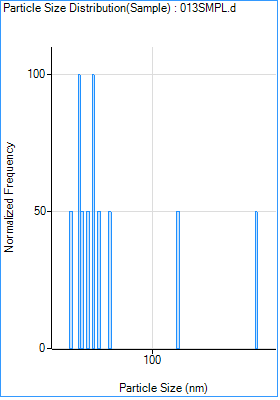

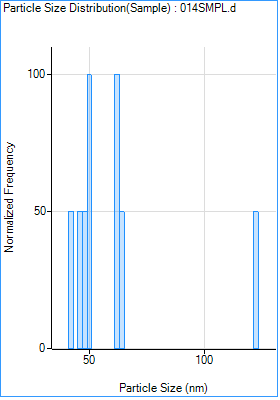


**D**

**C**

**B**

**A**

**F**

**E**

**G**

Fig S1 Particle size distributions from spICP-MS analysis for A) standard of 21nm TiO_2_ nanoparticles, B) sample P2, C) sample P4, D) sampleP5, E) sample P9, F) sample P20, G) sample P22
